# The effect of QTL-rich region polymorphisms identified by targeted DNA-seq on pig production traits

**DOI:** 10.1007/s11033-018-4170-3

**Published:** 2018-04-05

**Authors:** Anna Stuczyńska, Katarzyna Piórkowska, Mirosław Tyra, Kacper Żukowski

**Affiliations:** 1 Department of Animal Molecular Biology, National Research Institute of Animal Production, 32-083 Balice, Poland; 2 Department of Pig Breeding, National Research Institute of Animal Production, 32-083 Balice, Poland; 3 University of Huddersfield, School of Applied Science, CeBioR, Queensgate, HD1 3DH Huddersfield, Great Britain; 4 Department of Cattle Breeding, National Research Institute of Animal Production, 32-083 Balice, Poland

**Keywords:** Targeted DNA sequencing, Genetic marker, Pork quality, Fibronectin

## Abstract

The aim of the present study was to analyse the effect of *PLCD4, PECR, FN1* and *PNKD* mutations on pig productive traits and tested the usefulness of targeted enrichment DNA sequencing method as tool for preselection of genetic markers. The potential genetic markers for pig productive traits were identified by using targeted enrichment DNA sequencing of chromosome 15 region that is QTL-rich. The selected mutations were genotyped by using HRM, Sanger sequencing and PCR-ACRS methods. The association study was performed by using GLM model in SAS program and included over 500 pigs of 5 populations maintained in Poland. The variation (C/T) of *PLCD4* gene affected feed conversion, intramuscular fat and water exudation. The *PNKD* mutations were associated with texture parameters measured after cooking. In turn, the variation rs792423408 (C/T) in the *FN1* gene influenced toughness measured in *semimembranosus* muscle and growth traits that was observed particularly in Duroc breed. Summarizing, the investigated gene variants delivered valuable information that could be used during developing SNP microarray for genomic estimated breeding value procedure in pigs. Moreover, the study showed that the TEDNA-seq method could be used to preselect the molecular markers associated with pig traits. However, the further association study that included large number animal populations is necessary.

**Electronic supplementary material:**

The online version of this article (10.1007/s11033-018-4170-3) contains supplementary material, which is available to authorized users.

## Introduction

One of the approaches based on next-generation sequencing (NGS) is targeted enrichment of genomic DNA (TEDNA-seq) sequencing, which allows the identification of a directly selected genomic region by using hybridization probes during DNA library preparation. Compared with a whole-genome strategy, the cost and effort of TEDNA-seq analysis can be lower because of selective recovery and subsequent sequencing. Targeted enrichment sequencing is mainly applied to whole-exome analysis, which enables the identification of many diseases or metabolic pathways [1]. However, the bioinformatics of TEDNA-seq is a big challenge due to the complex analysis that includes choosing the most suitable software, algorithm, and filtering parameters to avoid false positive gene variant calls [2].

On the other hand, based on the current release of the Pig QTL database, there are 17 955 QTLs (quantitative trait loci)/associations identified from 576 publications that represent 635 different traits associated with meat and carcass traits, health, exterior, production and reproductive traits (http://www.animalgenome.org/cgi-bin/QTLdb/SS/index). In pigs, chromosome 15 contains a region between the microsatellites *SW1683* and *SW906* (79.3–89.3 cM, 127–135 Mbp) which is highly rich in QTLs associated with meat quality, fat content and growth traits. In this region, the abundance of QTLs related to meat quality traits, in particular meat texture, is the highest in comparison to other pig chromosomes. That SSC15 region contains more than 60 genes, including *ABCA12, PRKAG3, MARCH4, DES, CYP27A1, OBSL1, IGFBP2* and *IGFBP5*, which have been already investigated for their relationship with meat quality and growth traits [3]. The *PRKAG3* encode a muscle-specific isoform of the regulatory γ subunit of adenosine monophosphate-activated protein kinase (AMPK) [4]. A missense mutation in the *PRKAG3* gene was detected that affected the pH and meat colour in pigs [5]. In turn, the *DES* gene encodes a cytoskeletal protein called desmin, which is located in the outer periphery of the Z-disk in skeletal muscle fibres. This protein plays an important role in joining the neighbouring myofibrils and adhering them to the cell membrane [6]. Starkey et al. [7] postulate that there is a strong correlation between desmin degradation and increase in tenderness and water exudation in ovine meat products, but the desmin degradation was not researched in relation to pork. And the study of Piórkowska et al. [8] showed that the *ABCA12* promoter insertion affected daily gain, feed conversion and meat brightness. Although, that numerous genes located in the SSC15 region of interest has been analysed for their effect on pig traits, this region is still highly curious. Because, it probably contains unknown genetic markers associated with pork texture parameters and fat content.

Therefore, the aim of the present study was the analysis of *PECR, FN1, PNKD* and *PLCD4* mutation effect on pig productive traits with particular emphasis on pork texture parameters. These mutations are located in the region on chromosome 15 and they were selected using TEDNA-seq method. The preliminary study was conducted by using two pig breeds Polish Landrace and Puławska that are significantly different in fat content, meat quality and growth traits. In turn, the main association study was performed by using over 500 pigs belonging to 5 pig populations maintained in Poland.

## Materials and methods

### Animals

All animals used in the investigation originated from different farms, were female and unrelated. They were delivered to Pig Test Station of National Research Institute of Animal Production located in Chorzelów, Mełno, Pawłowice, and Rossocha. All pigs were maintained under the same environmental and diet condition according to Pig Test Station procedure. They were fed ad libitum by initial weight at 30 kg and finished at 100 ± 2 kg. The growth traits were measured during Test and the body composition, fat content and meat quality parameters were assessed after slaughter according to Tyra and Żak et al. [9] and meat texture parameters as was described by Ropka-Molik et al. [10].

### DNA isolation

The blood samples were collected during slaughter and stabilized by EDTA. DNA was isolated from whole blood by Sherlock AX (A&A Biotechnology) kit.

### Targeted enrichment DNA sequencing

The TEDNA-seq analysis, performed for another study [11], included 16 pigs of the Polish Landrace (n = 8) and Puławska (n = 8) breeds, which differ in growth, meat quality and fat content traits. The enrichment technique applied in this approach was RNA hybrid capture (1× tiling). The hybridization probes for the region of interest with exclusion of repeat-masked elements [12], which reduce the recovery of undesirable products, were designed by the Agilent team. Each library was indexed with a unique adaptor that enabled identification of most mutations for specific animals and then made it possible to perform an association study on chosen pigs. Filtered sequences were aligned to the *Sus scrofa* genome (Sscrofa10.2 assembly).

### Selection of potential biomarkers

After performing TEDNA-seq on 16 chosen pigs, the analysis provided information about all gene variants in the SSC15 region, our region of interest, which is QTL-rich. The preliminary association analysis was conducted using LRT test in R-project. The LRT test was used to compare likelihood of linear null model versus alternative mode with SNP fixed effect.

$${\text{LRT = }}-{\text{2lo}}{{\text{g}}_e}\left( {\frac{{{\mathcal{L}_s}(\hat {\theta })}}{{{\mathcal{L}_g}(\hat {\theta })}}} \right)$$

The difference in log-likelihoods was compared with a Chi-squared distribution. The selection criteria of gene variants for further analysis were: *P* value ≤ 0.05 of LRT test obtained for at least few investigated pig productive traits and the relationship of identified mutations with genes likely involved in shaping the pig phenotype. The functional analysis of genes having significant mutations was carried out by Panther, STRING and KEGG. All selected gene variants were validated by Sanger sequencing.

### Genotyping of selected mutations with the potential to become biomarkers

Five mutations with high potential were selected. The genotyping was performed for 535 pigs of 5 breeds: Puławska, Polish Landrace, Polish Large White, Pietrain and Duroc by using different molecular techniques.

The missense variant rs792423408 (C/T) of the *FN1* gene and the upstream gene variant rs343851532 (C/T) of the *PECR* gene were genotyped by the high-resolution melting (HRM) technique. The KAPA™ HRM FAST PCR Kit (Kapa Biosystems, USA) was used to perform the HRM method on a QuantStudio 7 Flex Real-Time PCR System (Thermo Scientific, USA). The mutation rs324680963 (C/T), located upstream of the *PLCD4* gene, was analysed by the PCR-ACRS method. PCR was performed using the AmpliTaq® 360 Gold Master Mix Kit (Thermo Scientific, USA) on a Mastercycler® Nexus Gradient (Eppendorf), and then the PCR product was digested in 37 °C with a mixture of 4U restriction enzyme *Rsa*I (New England Biolabs, USA). The missense rs329501722 (C/A) and the frameshift rs792243103 (-/C) in the first exon of the *PNKD* gene variants were sequenced by Sanger sequencing using the BigDye® Terminator v3.1 Sequencing Kit (Thermo Scientific, USA) on a 3500XL Genetic Analyzer (Applied Biosystems, USA).

### Statistical analysis

The gene variant effects on 535 gilts were estimated by using the GLM procedure (SASv.8.02). The final model was

$${{\text{Y}}_{{\text{ijk}}}}=\mu +{{\text{d}}_{\text{i}}}+{{\text{b}}_{\text{j}}}+{{\text{s}}_{\text{k}}}+{{\text{e}}_{{\text{ijk}}}}$$

where, Y_ijk_ is the observation, µ is the overall mean of the trait, d_i_ is the fixed effect of the k_th_ genotypes of gene, b_j_ is the fixed effect of breed, s_k_ is the fixed effect of pig station, e_ijk_ is the random error.

To estimate significance differences between means, the Least Mean Squares (LSM) test was used. All results are shown as LSM ± SE.

## Results

### The selection of interesting gene variants after targeted enrichment sequencing of DNA

By using LRT test and functional analysis five mutations of *PECR, FN1, PNKD* and *PLCD4* genes (Table 1) showing possible association with pig production traits were selected. The whole gene variant list with *P* values after LRT test is available by link goo.gl/dXenef.

**Table 1:** Chosen variation of genes that could be associated with pig production traits

ID number^a^	Position on SSC15^b^	Reference allele	Alternate allele	Localization or effect	Gene
rs324680963	133641482	C	T	Upstream gene variant	*PLCD4*
rs792423408	130416725	C	T	Missense variant	*FN1*
rs343851532	131216842	C	T	Upstream gene variant	*PECR*
rs329501722	133370271	C	A	Missense variant	*PNKD*
rs792243103	133370291	–	C	Frameshift variant	*PNKD*

^a^ID number of SNP database (NCBI)

^b^position on SSC15 according to Sscrofa 10.2 assembly

### Validation of significant gene variants by using the Sanger sequencing method

**Fig. 1:**
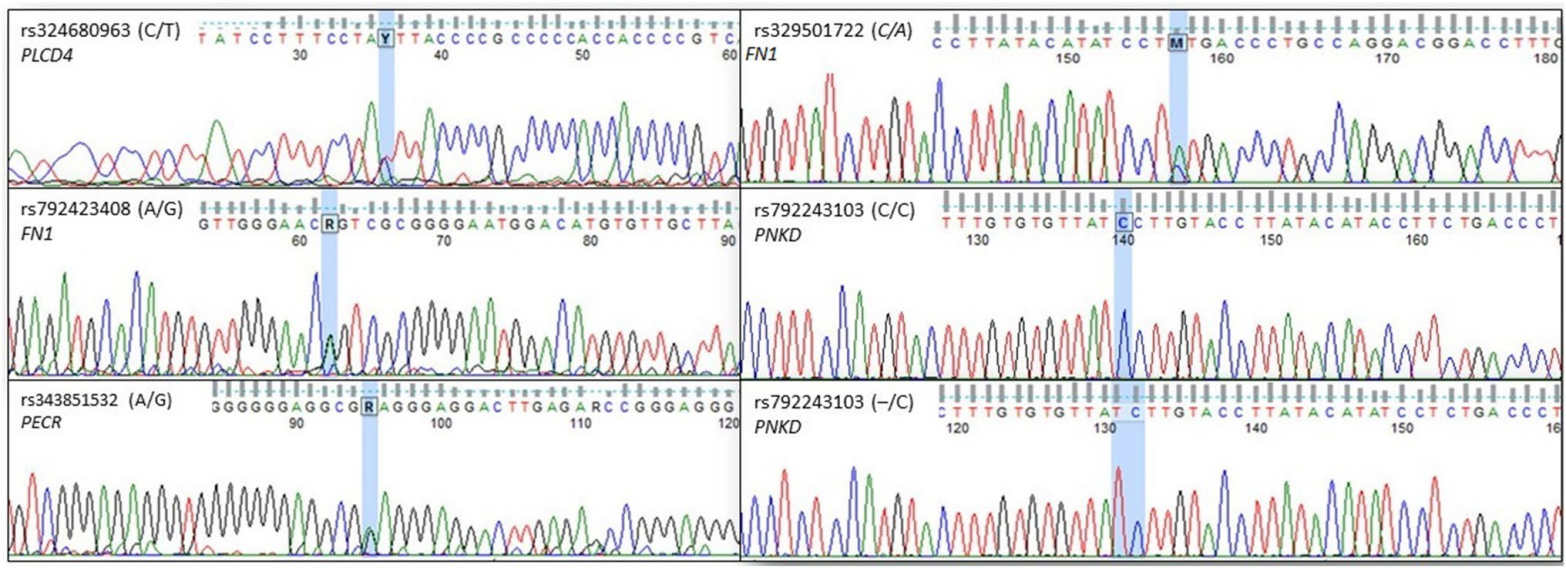
The chromatogram showing the variations of *PLCD4, FN1, PECR* and *PNKD* genes

The validation of identified significant gene variants was performed by Sanger sequencing method using a 3500XL Genetic Analyzer (Applied Biosystems, USA) to verify their position on porcine chromosome 15. Chromatograms that confirmed the presence of variations in chosen genes were shown in Fig. 1.

### The effect of significant gene variants on pig productive traits

#### PLCD4 gene

The PCR-ACRS method was used to genotype the mutation (rs324680963) in the gene *PLCD4*. In was observed that alternate T allele occurred with 13% frequency and homozygotes TT constituted only 4%. The highest number of pig individuals with the TT genotype has been observed in Polish Landrace (7%). In turn, in the Duroc, TT pigs were not present (Table S1).

The variation (C/T) of the *PLCD4* gene significantly influenced feed conversion (kg/kg), intramuscular fat (IMF) and water exudation. The individuals with the CC genotype presented the highest feed conversion values in comparison to pigs with the other genotypes. Moreover, TT Polish Landrace pigs showed higher IMF (*P* < 0.05) than the other investigated pigs, which revealed similar values of IMF. In turn, CC Polish Large White individuals characterized by the lowest values of water exudation (− 6 units, *P* < 0.05). This trend was observed for all breeds except Pietrain. The opposite trend was identified for an average backfat thickness, where TT pigs seemed to have thicker backfat (Table 2).

**Table 2:** LSM ± S.E. for chosen interesting pig traits by *PLCD4* genotypes, which presented the significant effect or trend

Traits	*Genotype PLCD4*	PLW	PUL	PL	DUR	PIET	Total^1^	GLM significance		
								*PLCD4*	Breed	Pig test station
Feed conversion (kg/kg)	*CC* *CT* *TT*	2.46^±0.07^ 2.48^±0.08^ 2.31^±0.10^	2.90^±0.02^ 2.94^±0.05^ 3.12^±0.25^	2.71^±0.03^ 2.79^±0.03^ 2.82^±0.07^	2.82^±0.04^ 2.99^±0.17^ –	2.89^±0.07^ 3.08^±0.12^ 3.13^±0.20^	2.80^±0.04^A 2.88^±0.05^B 2.88^±0.07^B	**	***	***
IMF	*CC* *CT* *TT*	1.24^±0.09^ 1.23^±0.10^ 1.18^±0.14^	1.16^±0.03^ 1.30^±0.07^ 1.04^±0.32^	1.18^±0.02^a 1.18^±0.03^a 1.32^±0.05^b	2.35^±0.07^ – –	2.18^±0.16^ 2.47^±0.29^ –	2.24^±0.07^ 2.36^±0.09^ 2.45^±0.13^	*	***	***
Water exudation	*CC* *CT* *TT*	30.4^±3.24^a 36.3^±3.57^b 33.2^±4.93^ab	33.5^±0.76^ 34.5^±1.46^ –	35.7^±0.75^ 37.8^±0.78^ 37.0^±1.81^	31.5^±0.83^ – –	32.5^±1.52^ 29.8^±3.08^ 26.8^±7.43^	32.8^±1.10^ 35.1^±1.29^ 33.9^±2.07^	**	***	***
Carcass yield	*CC* *CT* *TT*	77.0^±1.51^ 77.8^±1.64^ 78.3^±2.13^	75.6^±0.28^ 77.2^±0.63^ 73.2^±3.04^	76.2^±0.29^ 76.5^±0.33^ 77.5^±0.77^	75.8^±0.28^ 76.9^±1.14^ –	77.1^±0.60^ 76.7^±1.06^ 78.9^±1.85^	76.9^±0.47^ 77.5^±0.54^ 78.1^±0.82^	*	***	***
Backfat thickness	*CC* *CT* *TT*	1.65^±0.17^ 1.65^±0.18^ 1.76^±0.24^	1.58^±0.03^ 1.52^±0.07^ 2.20^±0.36^	1.24^±0.04^ 1.29^±0.04^ 1.35^±0.10^	1.49^±0.04^ 1.84^±0.15^ –	1.13^±0.06^ 1.15^±0.10^ 1.15^±0.17^	1.48^±0.05^ 1.50^±0.06^ 1.60^±0.09^	ns	***	***

Allele: *C* wild, *T* mutation. Values with the same superscripts belong to the same statistical group (A, B = *P* < 0.01, a, b = *P* < 0.05). *PLW* Polish Large White, *PL* Polish Landrace, *DUR* Duroc, *PIET* Pietrain, *IMF* intramuscular fat

^1^Means for pig traits independent on breed

#### FN1 gene

To genotype the variation rs792423408 (C/T) in the *FN1* gene, the HRM method was applied. It was observed that the majority of investigated pigs belonged to CC homozygotes, CT heterozygous genotype was identified in just 10% pigs and TT individuals were absent. And in the Pietrain, only one CC genotype was detected. The frequencies of the genotypes are shown in (Table S2).

The influence of the *FN1* gene on the toughness of cooked *semimembranosus* muscle was observed with a significance of *P* < 0.05 (Table 3). In all investigated breeds, the CT heterozygotes were characterized by higher values of toughness (+ 16 N/mm/s) than the CC homozygotes. The overall analysis showed a tendency that the CC pigs characterized by higher daily gain and achieved faster slaughter mass than CT pigs. Significant results were observed for the Duroc pigs, where CC pigs had 83 g higher daily gain (*P* < 0.05) and achieved a faster finish day (*P* < 0.01, 26 days earlier) than the CT Duroc pigs. Similarly in the Polish Landrace, where the CC pigs finished the Test 6 days faster than CT pigs (*P* < 0.05). In regards to meat quality traits, the CT Puławska pigs had higher values of both meat lightness and yellowness than the CC homozygotes (*P* < 0.05). Moreover, it was observed that rs792423408 (C/T) *FN1* mutation probably affected meat texture parameters. Significant results were detected in Polish Landrace where heterozygotes showed 15% higher values of toughness than the CC pigs (*P* < 0.01).

**Table 3:** LSM ± S.E. for chosen interesting pig traits by *FN1* genotypes, which presented the significant effect or trend

Traits	*Genotype FN1*	PLW	PUL	PL	DUR	PIET	Total^1^	GLM significance		
								*FN1*	Breed	Pig test station
Daily gain (g)	*CC* *CT*	608^±5^ 612^±19^	540^±5^ 545^±22^	612^±6^ 591^±10^	599^±9^a 516^±35^b	611^±12^ –	624^±10^ 609^±13^	0.09	***	***
Age of slaughter (days)	*CC* *CT*	167^±1^ 166^±6^	188^±2^ 181^±10^	166^±2^a 173^±3^b	173^±3^A 199^±10^B	173^±4^ –	169^±3^ 174^±4^	ns	***	***
ML Meat colour lightness	*CC* *CT*	54.1^±0.19^ 54.1^±1.13^	54.3^±0.22^a 57.2^±1.20^b	54.9^±0.26^ 55.2^±0.35^	54.1^±0.47^ –	53.8^±0.72^ –	54.0^±0.42^ 54.6^±0.58^	ns	**	***
MB Meat colour yellowness	*CC* *CT*	2.27^±0.07^ 2.58^±0.42^	2.40^±0.07^a 3.28^±0.35^b	2.32^±0.07^ 2.43^±0.10^	3.38^±0.19^ –	4.59^±0.52^ –	4.34^±0.20^ 4.60^±0.27^	ns	***	***
Carcass yield (%)	*CC* *CT*	76.9^±0.26^ 77.8^±1.10^	76.1^±0.26^ 73.3^±1.15^	76.3^±0.25^ 76.9^±0.40^	75.8^±0.29^ 75.8^±1.15^	77.02^±0.57^ –	77.0^±0.47^ 77.1^±0.62^	ns	***	***
Weight of loin (kg)	*CC* *CT*	62.1^±0.26^ 60.4^±1.09^	59.6^±0.30^ 60.1^±1.32^	61.8^±0.31^ 61.6^±0.49^	61.9^±0.39^ 59.1^±1.53^	67.6^±0.52^ –	61.9^±0.52^ 61.5^±0.67^	ns	***	ns
Toughness of cooked *semimembranosus* muscle (N)	*CC* *CT*	220.5^±6.41^ 194.4^±16.0^	187.7^±3.87^ 209.4^±13.6^	188.6^±4.41^A 220.9^±10.7^B	197.2^±8.34^ 229.5^±30.4^	213.4^±6.90^ –	199.9^±2.64^a 216.4^±7.87^b	*	***	ns
Resilience of cooked *semimembranosus* muscle	*CC* *CT*	0.25^±0.003^ 0.24^±0.01^	0.25^±0.004^ 0.25^±0.02^	0.26^±0.004^ 0.25^±0.004^	0.27^±0.009^ 0.26^±0.008^	0.27^±0.005^ –	0.25^±0.002^ 0.25^±0.004^	ns	ns	***

Allele: *C* wild, *T* mutation. Values with the same superscripts belong to the same statistical group (A. B = *P* < 0.01. a. b = *P* < 0.05). *PLW* polish white large, *PL* polish landrace, *DUR* Duroc, *PIET* Pietrain

^1^Means for pig traits independent on breeds

#### PECR gene

The upstream *PECR* rs343851532 (C/T) variant was genotyped by the same as *FN1* mutation the HRM method. The analysis of five Polish pig breeds showed low frequency of C allele (13%) in these populations. In all breeds only two genotypes TT and CT were observed (Table S3).

The conducted association study showed that *PECR* rs343851532 (C/T) mutation affected meat content. The CC pigs characterized by higher carcass yield and weight of loin than the CT individuals. It was particularly observed in the Duroc pigs, where CC Duroc showed 300 g higher weight of loin than those with the CT genotype. In turn, in the Pietrain breed CC pigs presented 2% higher meat percentage than the heterozygotes (Table 4). In regards to meat texture parameters just tendencies were identified.

**Table 4:** LSM ± S.E. for chosen interesting pig traits by *PECR* genotypes, which presented the significant effect

Traits	*Genotype PECR*	PLW	PUL	PL	DUR	PIET	Total^1^	GLM significance		
								*PECR*	Breed	Pig test station
Carcasss yield (%)	*CC* *CT*	77.6^±0.31^A 75.8^±0.42^B	75.8^±0.29^ 76.3^±0.55^	76.2^±0.24^a 77.3^±0.45^b	75.9^±0.31^ 75.4^±0.55^	76.7^±0.64^ 77.5^±0.74^	77.0^±0.48^ 76.9^±0.51^	ns	***	***
Weigth of loin (kg)	*CC* *CT*	6.05^±0.07^a 5.81^±0.10^b	5.66^±0.06^ 5.72^±0.11^	5.94^±0.06^ 6.05^±0.11^	6.11^±0.06^A 5.77^±0.11^B	6.56^±0.12^ 6.64^±0.14^	6.43^±0.10^ 6.38^±0.11^	ns	***	***
Meat percentage (%)	*CC* *CT*	61.6^±0.53^ 62.6^±0.56^	59.7^±0.33^ 59.7^±0.62^	61.8^±0.30^ 61.7^±0.57^	61.7^±0.45^ 62.1^±0.78^	68.1^±0.59^a 66.3^±0.69^b	61.8^±0.53^ 61.8^±0.56^	ns	***	ns
Toughness of cooked *semimembranosus* muscle (N)	*CC* *CT*	217.0^±7.89^ 223.7^±10.2^	191.3^±4.38^ 179.5^±8.38^	194.4^±4.52^ 202.8^±11.8^	199.6^±9.53^ 206.2^±17.7^	212.0^±8.68^ 209.4^±10.0^	200.2^±2.86^ 204.0^±5.23^	ns	***	ns
Chewiness of cooked semimembranosus muscle	*CC* *CT*	4.71^±0.32^ 5.08^±0.37^	4.22^±0.21^ 3.74^±0.48^	4.30^±0.26^ 4.05^±0.38^	5.17^±0.57^ 4.24^±1.08^	6.26^±0.36^ 6.22^±0.77^	4.66^±0.14^ 4.62^±0.24^	ns	***	ns

Allele: *C* wild, *T* mutation. Values with the same superscripts belong to the same statistical group (A, B = *P* < 0.01, a, b = *P* < 0.05). *PLW* Polish Large White, *PL* Polish Landrace, *DUR* Duroc, *PIET* Pietrain

^1^Means for pig traits independent on breeds

#### PNKD gene

The variations rs329501722 (C/A) and rs792243103 (–/C) of the *PNKD* gene were genotyped using Sanger sequencing on a 3500 Genetic Analyzer (Applied Biosystems, USA) (Fig. 1).

The alternate A allele frequency of rs329501722 *PNKD* mutation was low and accounted only 13%. The 5% of investigated pigs revealed AA genotype and 24% belonged to heterozygotes (Table S4).

The association analysis showed that the *PNKD* rs329501722 (C/A) mutation influenced feed conversion, water exudation, resilience, cohesiveness for *semimembranosus* (ham) muscle and weight of loin. All investigated AA pigs showed higher water exudation (*P* < 0.01), resilience and cohesiveness values measured in cooked *semimembranosus*, and higher weight of loin (approx. 200 g, *P* < 0.05). It was particularly observed in PLW and PUL pigs, where AA pigs characterized by 5 and 10 units higher values of water exudation (*P* < 0.01) than the CC individuals. In the Duroc and Pietrain breeds the AA pigs were absent. Nevertheless, in the Duroc was observed that AC pigs showed lower values of daily gains (close to 100 g, *P* < 0.05) than the CC pigs. In the Polish Landrace, opposite tendencies were observed. The AA PL pigs exhibited the lowest growth performance parameters such as the 70 g lower daily gain (*P* < 0.05) and higher feed/gain ratio (*P* < 0.01), but their meat had higher IMF content (*P* < 0.05) than pigs with the other genotypes (Table 5).

**Table 5:** LSM ± S.E. for chosen interesting pig traits by *PNKD* genotypes, which presented the significant effect

Traits	*Genotype PNKD*	PLW	PUL	PL	DUR	PIET	Total^1^	GLM significance		
								*PNKD*	Breed	Pig test station
Daily gain in test (g)	*AA* *CA* *CC*	960^±43^ 907^±22^ 917^±9^	807^±28^ 799^±14^ 788^±8^	832^±28^b 903^±12^a 899^±13^	– 809^±54^b 914^±16^a	– 840^±29^ 838^±17^	869^±24^ 887^±18^ 886^±16^	ns	***	**
Feed conversion (kg/kg)	*AA* *CA* *CC*	2.64^±0.06^ 2.70^±0.03^ 2.66^±0.01^	2.76^±0.08^ 2.91^±0.04^ 2.92^±0.02^	2.94^±0.07^A 2.74^±0.03^B 2.72^±0.03^B	– 3.01^±0.15^ 2.80^±0.04^	– 3.09^±0.11^ 2.91^±0.06^	2.86^±0.06^ 2.84^±0.05^ 2.80^±0.04^	**	***	***
Days in test (days)	*AA* *CA* *CC*	75^±4^ 80^±2^ 78^±1^	88^±3^ 88^±2^ 89^±1^	87^±3^a 79^±1^b 80^±1^b	– 87^±5^ 79^±1^	– 88^±3^ 89^±2^	87^±2^ 84^±2^ 84^±2^	ns	***	0.06
IMF	*AA* *CA* *CC*	1.26^±0.09^ 1.21^±0.05^ 1.21^±0.02^	1.28^±0.12^ 1.19^±0.06^ 1.17^±0.04^	1.31^±0.05^a 1.17^±0.03^b 1.17^±0.02^b	– – 2.34^±0.08^	– 2.09^±0.35^ 2.26^±0.15^	2.31^±0.11^ 2.21^±0.09^ 2.20^±0.07^	ns	***	***
Water exudative	*AA* *CA* *CC*	39.7^±3.23^AB 40.6^±1.56^A 35.0^±0.67^B	42.5^±2.86^A 32.5^±1.23^B 32.9^±0.84^B	38.3^±1.66^ 37.1^±0.76^ 35.9^±0.83^	– – 31.3^±0.86^	– 28.1^±4.32^ 31.9^±1.38^	37.5^±1.80^A 34.2^±1.30^AB 32.6^±1.12^B	***	***	***
Toughness of cooked *semimembranosus* muscle (N)	*AA* *CA* *CC*	218.5^±25.0^ab 187.5^±13.4^b 221.3^±6.47^a	173.6^±8.26^ 188.2^±7.60^ 187.8^±4.51^	197.7^±12.5^ 204.5^±7.49^ 189.4^±5.57^	– 221.7^±26.3^ 199.2^±8.30^	– 214.0^±11.7^ 205.5^±8.67^	207^±38^ 213^±101^ 211^±52^	ns	***	ns
Resilience of cooked *semimembranosus* muscle	*AA* *CA* *CC*	0.24^±0.02^ 0.26^±0.007^ 0.25^±0.004^	0.30^±0.02^A 0.25^±0.005^B 0.24^±0.004^B	0.27^±0.02^ 0.26^±0.005^ 0.26^±0.005^	– 0.27^±0.02^ 0.26^±0.009^	– 0.28^±0.01^ 0.27^±0.006^	0.30^±0.05^A 0.28^±0.04^AB 0.27^±0.04^B	**	ns	***
Cohesiveness of cooked *semimembranosus* muscle	*AA* *CA* *CC*	0.60^±0.002^ 0.61^±0.01^ 0.60^±0.006^	0.67^±0.02^A 0.62^±0.01^AB 0.60^±0.08^B	0.64^±0.008^ 0.62^±0.008^ 0.62^±0.005^	– 0.64^±0.03^ 0.62^±0.01^	– 0.67^±0.01^ 0.64^±0.01^	0.67^±0.07^a 0.65^±0.06^ab 0.64^±0.07^b	*	ns	***
Weigth of loin (kg)	*AA* *CA* *CC*	6.32^±0.31^ 6.27^±0.16^ 5.94^±0.07^	5.87^±0.21^ 5.73^±0.11^ 5.63^±0.06^	6.21^±0.17^ 5.98^±0.08^ 5.88^±0.08^	– 6.21^±0.24^ 6.07^±0.07^	– 6.32^±0.18^ 6.62^±0.10^	6.72^±0.16^a 6.50^±0.12^ab 6.42^±0.10^b	*	***	***
Backfat thickness (cm)	*AA* *CA* *CC*	1.26^±0.15^ 1.29^±0.08^ 1.27^±0.03^	1.59^±0.13^ 1.46^±0.07^ 1.61^±0.04^	1.30^±0.09^ 1.27^±0.04^ 1.27^±0.04^	– 1.72^±0.13^ 1.50^±0.04^	– 1.30^±0.09^a 1.13^±0.05^b	1.48^±0.09^ 1.47^±0.06^ 1.47^±0.05^	ns	***	***
Primary cuts (kg)	*AA* *CA* *CC*	24.2^±0.58^ 24.4^±0.30^ 24.1^±0.13^	23.2^±0.57^ 23.1^±0.30^ 22.5^±0.16^	24.3^±0.33^ 23.9^±0.15^ 23.7^±0.15^	– 22.6^±0.64^ 22.8^±0.19^	– 26.0^±0.53^b 27.0^±0.30^a	25.3^±0.37^ 24.9^±0.28^ 24.8^±0.24^	ns	***	***

Allele: *C* wild, *A* mutation. Values with the same superscripts belong to the same statistical group (A. B = *P* < 0.01. a. b = *P* < 0.05). *PLW* polish large white, *PL* polish landrace, *DUR* Duroc, *PIET* Pietrain, *IMF* intramuscular fat

^1^Means for pig traits independent on breeds

The second identified in *PNKD* gene rs792243103 (–/C) mutation was more frequent. In investigated pig populations prevailed heterozygotes (46%) and the C deletion in both alleles occurred with 17% frequency (Table S5). The present study showed that this INDEL mutation affected the growth traits particularly in PUL pigs, which belong to native breeds and were not under selective pressure. In the PUL breed, the absence of C in position 202 of the *PNKD* transcript caused a slight decrease in daily feed intake. Those pigs were also characterized by higher toughness values measure in the *semimembranosus* muscle (*P* < 0.05) and resilience parameters. Moreover, this mutation affected meat yellowness and carcass yield (*P* < 0.05). The pigs with C insertions in both alleles presented higher values of meat yellowness (*P* < 0.05) and carcass yield and lower toughness values (− 12 N/mm/s, *P* < 0.05) than pigs with other genotypes. The CC PL individuals showed the lowest carcass yield (− 3%, *P* < 0.05), weight of loin (− 300 g, *P* < 0.05) and primer cuts (− 700 g, *P* < 0.05). On the other hand, in the CC Pietrain pigs significantly lower pH value measured in *longissimus dorsi* muscle in comparison to –/– pigs (Table 6).

**Table 6:** LSM ± S.E. for chosen interesting pig traits by rs792243103 (–/C) *PNKD* genotypes, which presented the significant effect or trend

Traits	*Genotype PNKD*	PLW	PUL	PL	DUR	PIET	Total^1^	GLM significance		
								*PNKD*	Breed	Pig test station
Daily feed intake (kg)	*–*/*–* *–*/*C* *CC*	2.40^±0.06^ 2.44^±0.04^ 2.45^±0.03^	2.20^±0.04^a 2.30^±0.02^b 2.32^±0.03^b	2.48^±0.04^ 2.44^±0.03^ 2.43^±0.04^	2.71^±0.24^ 2.49^±0.06^ 2.56^±0.05^	2.49^±0.08^ 2.43^±0.06^ 2.39^±0.07^	2.46^±0.05^ 2.46^±0.04^ 2.47^±0.04^	ns	***	***
MB Meat colour yellowness	*–*/*–* *–*/*C* *CC*	2.56^±0.23^ab 2.02^±0.12^b 2.41^±0.09^a	2.17^±0.19^ 2.51^±0.10^ 2.46^±0.11^	2.47^±0.11^ 2.29^±0.09^ 2.35^±0.14^	– 3.82^±0.35^ 3.21^±0.26^	5.59^±0.85^ 3.83^±0.67^ 4.89^±0.74^	4.65^±0.25^a 4.22^±0.22^b 4.46^±0.21^ab	*	**	***
*Longissimus dorsi* pH after 45 min	*–*/*–* *–*/*C* *CC*	6.31^±0.04^ 6.32^±0.02^ 6.30^±0.02^	6.28^±0.03^ 6.26^±0.02^ 6.32^±0.02^	6.35^±0.03^ 6.31^±0.02^ 6.25^±0.03^	6.70^±0.25^ 6.39^±0.07^ 6.42^±0.06^	6.41^±0.10^a 6.37^±0.09^a 6.17^±0.09^b	6.47^±0.04^ 6.43^±0.04^ 6.41^±0.04^	ns	***	***
Toughness of raw *semimembranosus* muscle (N)	*–*/*–* *–*/*C* *CC*	73.2^±6.54^ 77.0^±3.46^ 83.9^±2.67^	60.4^±4.38^a 90.9^±8.75^b 85.9^±7.91^ab	73.8^±3.29^ 73.7^±2.89^ 69.2^±4.33^	– 118.0^±18.9^ 106.0^±22.6^	74.2^±8.26^ 83.8^±5.08^ 71.0^±4.89^	88.6^±20.7^a 100.1^±47.8^b 97.3^±37.2^ab	ns	***	**
Cohesiveness of cooked *semimembranosus* muscle (N)	*–*/*–* *–*/*C* *CC*	0.61^±0.02^ 0.61^±0.01^ 0.61^±0.006^	0.63^±0.02^ 0.60^±0.008^ 0.60^±0.01^	0.62^±0.01^ 0.62^±0.006^ 0.62^±0.02^	– 0.62^±0.02^ 0.63^±0.02^	0.65^±0.02^ 0.64^±0.02^ 0.63^±0.02^	0.65^±0.07^ 0.64^±0.06^ 0.64^±0.07^	ns	ns	***
Resilience of cooked *semimembranosus* muscle (N)	*–*/*–* *–*/*C* *CC*	0.25^±0.01^ 0.25^±0.006^ 0.25^±0.004^	0.27^±0.01^a 0.24^±0.005^b 0.24^±0.006^b	0.26^±0.007^ 0.26^±0.004^ 0.25^±0.009^	– 0.26^±0.01^ 0.26^±0.01^	0.28^±0.01^ 0.28^±0.007^ 0.27^±0.008^	0.28^±0.04^ 0.27^±0.04^ 0.27^±0.04^	ns	ns	***
Carcass yield (%)	*–*/*–* *–*/*C* *CC*	76.7^±0.83^ 77.7^±0.48^ 76.8^±0.34^	76.9^±0.63^ 75.3^±0.37^ 76.1^±0.45^	77.5^±0.39^a 76.2^±0.29^a 75.8^±0.44^b	74.7^±1.96^ 75.2^±0.48^ 76.2^±0.41^	77.5^±0.85^ 77.3^±0.67^ 76.5^±0.77^	77.7^±0.56^a 76.9^±0.51^ab 76.9^±0.49^b	*	***	***
Weight of loin (kg)	*–*/*–* *–*/*C* *CC*	5.92^±0.19^ 6.13^±0.11^ 5.95^±0.08^	5.69^±0.12^ 5.60^±0.07^ 5.74^±0.09^	6.16^±0.10^a 5.91^±0.07^ab 5.79^±0.11^b	5.71^±0.45^ 6.11^±0.11^ 6.06^±0.10^	6.57^±0.15^ 6.73^±0.12^ 6.43^±0.14^	6.52^±0.12^ 6.47^±0.11^ 6.39^±0.10^	ns	***	***
Primary cuts (kg)	*–*/*–* *–*/*C* *CC*	24.0^±0.36^ 24.4^±0.20^ 24.0^±0.15^	22.9^±0.34^ 22.5^±0.20^ 22.8^±0.24^	24.2^±0.18^a 23.8^±0.14^ab 23.5^±0.21^b	23.0^±1.21^ 23.3^±0.30^ 24.1^±0.26^	27.1^±0.46^ 26.8^±0.37^ 27.0^±0.42^	25.1^±0.28^ 24.8^±0.25^ 24.8^±0.25^	ns	***	***

Allele: *C* wild, *- * deletion. Values with the same superscripts belong to the same statistical group (A, B = *P* < 0.01; a.,b = *P* < 0.05). *PLW* Polish Large White, *PL* Polish Landrace, *DUR* Duroc, *PIET* Pietrain

^1^Means for pig traits independent on breeds

## Discussion

The present study examined the targeted enrichment DNA sequencing method as tool for preselection of potential genetic markers. The positive results of the analysis could lead to the discovery of new possible ways to use this novel, fast and cost-effective technique. By using this method and functional analysis, five potential genetic markers were selected and included into downstream association study to show their effects on porcine productive traits.

In position  − 123 bp of the pig *PLCD4* gene, one mutation (rs324680963) was identified that can introduce changes to the *PLCD4* promoter region or in the sequence that enhances or silences gene expression. The analysis using the PROMO3 freeware showed that this mutation can affect the binding of numerous transcription factors (TFs). The *PLCD4* gene encodes phospholipase C delta 4, which activates Ca^2+^ ions during the leptin signalling pathway and induces the activity of NPY neurons and possibly affects feed intake. Additionally, phospholipase C participates indirectly in the thyroid hormone signalling pathway, which controls the metabolism of carbohydrates, proteins and lipids, thus having a potential influence on the growth traits [13]. In the present study, the upstream gene variant in the *PLCD4* gene showed a significant impact on the feed conversion, IMF, water exudation and carcass yield. The CC homozygotes presented lower feed conversion than the pigs with other genotypes. However, the influence on feed intake was not observed. These results suggest that the identified mutation has not altered *PLCD4* expression or that the phospholipase C delta 4 does not play such an important role in leptin signalling pathways, which was reflected in the phenotype. Nevertheless, it was shown that there is some significance in relation to feed conversion that is connected to the metabolism rate, thus implicating that this gene could play a meaningful role in the regulation of metabolism via the thyroid signalling pathway. Such a hypothesis could be explained by the research carried out by Onteru et al. [14] that showed the relationship between lipid metabolism and residual feed intake in Yorkshire pigs. In turn, Piórkowska et al. [15] proposed the *PLCD4* gene as a candidate gene for meat quality traits such as IMF and fat content because of the probable connection with fatty acid metabolism. Our research also showed a high impact of the *PLCD4* gene on IMF and water exudation, which should be further developed in studies included other pig breeds.

Another interesting gene variant that was located  − 23 bp upstream of the *PECR* gene seemed to be highly promising and was tested in the present study as a potential biomarker. Because the analysis of predicting the transcription factor binding sites by PROMO3 determined that the replacement of cytosine to thymine causes the loss of binding sites for 14 transcription factors and generates new binding sites for 5 novel TFs, this variant should significantly affect the *PECR* gene expression. The *PECR* encodes peroxisomal trans-2-enoyl-CoA reductase that is involved in fatty acid chain elongation and biosynthesis of unsaturated fatty acids. This reductase has high affinity to fatty acids with chain lengths up to 16 carbons [16]. A recent study by Huttlin et al. [17] postulated that PECR interacts with actin alpha 2 (ACTA2), which is involved in actin cytoskeleton and calcium signalling pathways, among others. It is possible that PECR, by interacting with ACTA2, can indirectly influence the growth traits, or more likely, it can have an impact on meat quality traits such as IMF and fat content because of its main function. This hypothesis was also confirmed by Sadkowski et al. [18], who researched novel genes that can be correlated with higher IMF. They found that the *PECR* gene is one of the novel genes that can be implicated in beef marbling. Another study supporting the participation of the *PECR* gene in fat deposition was carried out by Kärst et al. [19], where they investigated the effect of the *PECR* gene on shaping the intramuscular fat level and water holding capacity values in mice with high muscle mass. In turn, Piórkowska et al. [20] analysed the *PECR*145T>C mutation effect on pork quality in Polish pig breeds and found thicker backfat, lower loin intramuscular fat content, lower texture parameter values, and higher *PECR* expression measured in the *longissimus dorsi* muscle in pigs with the TT genotype. Therefore, they recommended the *PECR* gene as one of the candidate genes influencing fat mass. In the present study,  − 23 bp upstream of the *PECR* gene mutation affected only meat content traits. The effect on fat or IMF content were not observed. It suggests that this mutation does not regulate *PECR* expression. However, according to the latest pig genome release (Sscrofa11.1 assembly), this mutation is located also in 5` upstream regions of *XRCC5* and *FAM169* genes, therefore it could be associated with controlling their expression. Nevertheless, the abundance of publications with significant results focused on the function of the *PECR* gene in fat deposition in different animals suggests that the *PECR* polymorphisms could negatively influence pig productive traits by increasing the fat content. Therefore *PECR* mutations should be further investigated.

Another gene variation investigated in the present study was a missense variant in the *FN1* gene (rs792423408). This type of mutation is highly interesting because it leads to an alteration in the amino acid sequence, which could influence the protein function. The SIFT tool, available in the Ensembl database, predicted that the identified missense mutation belongs to a deleterious gene variant located in the DNA sequence encoding important protein domains. The *FN1* gene encodes fibronectin 1, which is involved in multiple pathways in the organism, the most interesting of which are the integrin pathway, extracellular matrix-receptor interaction, and regulation of actin cytoskeleton. The main function of fibronectin 1 is to bind cell surfaces and various compounds including collagen, fibrin, and actin as they participate in creating the extracellular matrix and basement membrane [21]. Fibronectins take part in cell adhesion and motility but also in opsonization, wound healing, and maintenance of cell shape [22, 23]. In the present study, it was observed that the missense variant of the *FN1* gene influenced the toughness values measured in cooked *semimembranosus* muscle (*P* < 0.05). In turn, Cassar-Malek et al. [24] investigated the effect of myostatin in double-muscled cattle, and its connection with *FN1* indicated that the fibronectin 1 gene is down-regulated in double-muscled cattle. This result suggests that *FN1* expression is switched off when massive muscle growth occurs. Moreover, Ponsuksili et al. [25], searching for the genes that can influence the water holding capacity in Duroc and Pietrain pigs, found that the *FN1* gene has a negative impact on water holding capacity, which can indicate that this protein has an essential role in maintaining the tight structure of muscles. However, in the present study, similar *FN1* gene effects were not observed.

The last gene variations that were investigated in the present study were missense and frameshift variants of the *PNKD* gene. However, according to the latest pig genome release (*SusScrofa* assembly 11.1) it was shown that both polymorphisms are located in intron region of *PNKD* gene. Thus, they are rather involved in the regulation of *PNKD* expression than they alter amino acid sequence. Nevertheless, the function of the *PNKD* gene is still unknown, but there are some assumptions that the protein encoded by this gene could be a possible hydrolase [23]. However, STRING v.10 predicted a connection between the PNKD and MAPK3, ENO3, MYL12B, ILK and UBC proteins. MAPK3 participates in the MAPK/ERK pathway, which is essential for muscle cell proliferation and regeneration [26]. ENO3 is a protein called enolase 3 that is involved in muscle development in animals [27]. ILK, or integrin-linked kinase, takes part in the mediation of protein–protein interactions, thus connecting the ECM with intracellular cytoskeleton and signalling proteins [28]. In turn, UBC is a protein called ubiquitin C that participates principally in protein degradation. As shown above, all proteins that potentially interact with PNKD are connected in different ways with muscle growth. In the present study, It was shown that rs329501722 variant affected water exudation, weight of loin, ham resilience and cohesiveness (*P* < 0.01), which was particularly apparent in Puławska pigs, a breed that was not under selective pressure. The pigs with the CC genotype showed lower water exudation and better texture parameters; thus, selection for this allele can affect the pork quality. In turn, the frameshift variant of *PNKD* seems to influence meat yellowness, ham resilience and meat content traits (*P* < 0.05). Moreover, in the Polish Landrace breed, the effect on weight of loin, primary cuts and carcass yield was observed. Unfortunately, no publication has been written about the *PNKD* gene and its effect on any pig production traits, which makes it difficult to compare the results. However, in this manuscript were shown the interesting dependency between *PNKD* mutations and pig productive traits, thus *PNKD* effects should be further studied.

To summarize, the targeted enrichment DNA sequencing method could be used to preselection of molecular markers, but downstream association studies including large number animal populations are necessary. On the other hand, the investigated gene variants provided valuable information that could be used for the development of SNP microarrays to improve genomic estimated breeding value procedures in pigs.

## Electronic supplementary material

Below is the link to the electronic supplementary material.

Supplementary material 1 (DOCX 42 KB)
