# Supplementary material for: The effect of QTL-rich region polymorphisms identified by targeted DNA-seq on pig production traits
Source: Mol Biol Rep. 2018 Apr 5;45(3):361–71. doi: 10.1007/s11033-018-4170-3 (PMC5966500; doi:10.1007/s11033-018-4170-3)
Supplement: Supplementary file 1 — Supplementary material 1 (DOCX 42 KB) [file 11033_2018_4170_MOESM1_ESM.docx]

| **Table S1.** The frequencies of alleles and genotypes of rs324680963 (C/T) in *PLCD4* gene in 5 Polish breeds. | | | | | | |
| --- | --- | --- | --- | --- | --- | --- |
| Pig Breed | Frequencies of rs792423408 (C/T) in *PLCD4* gene | | | | | |
|  | Genotype | | | Allele | | HWE*  (*P*-value) |
|  | CC | CT | TT | C | T |  |
| Polish Landrace | 0.51 (69) | 0.42 (55) | 0.07 (10) | 0.72 | 0.28 | 0.83 |
| Polish Large White | 0.81 (107) | 0.16 (21) | 0.03 (4) | 0.89 | 0.11 | 0.03 |
| Puławska | 0.83 (122) | 0.16 (23) | 0.01 (1) | 0.91 | 0.09 | 0.94 |
| Duroc | 0.94 (49) | 0.06 (3) | 0.00 (0) | 0.97 | 0.03 | 0.83 |
| Pietrain | 0.84 (74) | 0.13 (11) | 0.03 (3) | 0.90 | 0.10 | 0.01 |
| Total | 0.76 (421) | 0.20 (113) | 0.04 (18) | 0.87 | 0.13 | 0.003 |
| *HWE – Hardy-Weinberg Equilibrium: If p-value < 0.05 - not consistent with HWE, Not accurate if <5 individuals in any genotype group. In brackets number of individuals in genotype group. | | | | | | |

| **Table S2.** The frequencies of genotypes of rs792423408 (C/T) in *FN1* gene in 5 Polish breeds. | | | | | | |
| --- | --- | --- | --- | --- | --- | --- |
| Pig Breed | Frequencies of rs792423408 (C/T) in *FN1* gene | | | | | |
|  | Genotype | | | Allele | | HWE*  (*P*-value) |
|  | CC | CT | TT | C | T |  |
| Polish Landrace | 0.71 (94) | 0.29 (38) | 0.00 (0) | 0.86 | 0.14 | 0.053 |
| Polish Large White | 0.95 (124) | 0.05 (7) | 0.00 (0) | 0.97 | 0.03 | 0.75 |
| Puławska | 0.95 (135) | 0.05 (7) | 0.00 (0) | 0.98 | 0.02 | 0.76 |
| Duroc | 0.94 (48) | 0.06 (3) | 0.00 (0) | 0.97 | 0.03 | 0.83 |
| Pietrain | 1.00 (92) | 0.00 (0) | 0.00 (0) | 1.00 | 0.00 | 0.00 |
| Total | 0.90 (493) | 0.10 (55) | 0.00 (0) | 0.95 | 0.05 | 0.22 |
| *HWE – Hardy-Weinberg Equilibrium: If p-value < 0.05 - not consistent with HWE, Not accurate if <5 individuals in any genotype group. In brackets number of individuals in genotype group. | | | | | | |

| **Table S3.** The frequencies of alleles and genotypes of rs343851532 (C/T) in *PECR* gene in 5 Polish breeds. | | | | | | |
| --- | --- | --- | --- | --- | --- | --- |
| Pig Breed | Frequencies of rs343851532 (C/T) in *PECR* gene | | | | | |
|  | Genotype | | | Allele | | HWE*  (*P*-value) |
|  | CC | CT | TT | C | T |  |
| Polish Landrace | 0.78 (102) | 0.22 (28) | 0.00 (0) | 0.89 | 0.11 | 0.17 |
| Polish Large White | 0.65 (83) | 0.35 (44) | 0.00 (0) | 0.83 | 0.17 | 0.02 |
| Puławska | 0.78 (112) | 0.22 (32) | 0.00 (0) | 0.89 | 0.11 | 0.13 |
| Duroc | 0.74 (37) | 0.26 (13) | 0.00 (0) | 0.87 | 0.13 | 0.29 |
| Pietrain | 0.76 (72) | 0.24 (23) | 0.00 (0) | 0.88 | 0.12 | 0.18 |
| Total | 0.74 (406) | 0.26 (140) | 0.00 (0) | 0.87 | 0.13 | 0.00 |
| *HWE – Hardy-Weinberg Equilibrium: If p-value < 0.05 - not consistent with HWE, Not accurate if <5 individuals in any genotype group. In brackets number of individuals in genotype group. | | | | | | |

| Table S4. The frequencies of alleles and genotypes of rs329501722 (C/A) in *PNKD* gene in 5 Polish breeds. | | | | | | |
| --- | --- | --- | --- | --- | --- | --- |
| Pig Breed | Frequencies of rs329501722 (C/A) in *PNKD* gene | | | | | |
|  | Genotype | | | Allele | | HWE*  (*P*-value) |
|  | CC | AC | AA | C | A |  |
| Polish Landrace | 0.45 (59) | 0.46 (61) | 0.09 (12) | 0.68 | 0.32 | 0.50 |
| Polish Large White | 0.81 (107) | 0.15 (19) | 0.04 (5) | 0.89 | 0.11 | 0.00 |
| Puławska | 0.72 (98) | 0.22 (30) | 0.06 (8) | 0.83 | 0.17 | 0.01 |
| Duroc | 0.92 (45) | 0.08 (4) | 0.00 (0) | 0.96 | 0.04 | 0.77 |
| Pietrain | 0.83 (75) | 0.17 (15) | 0.00 (0) | 0.92 | 0.08 | 0.39 |
| Total | 0.71 (384) | 0.24 (129) | 0.05 (25) | 0.83 | 0.17 |  |
| *HWE – Hardy-Weinberg Equilibrium: If p-value < 0.05 - not consistent with HWE, Not accurate if <5 individuals in any genotype group. In brackets number of individuals in genotype group. | | | | | | |

| Table S5. The frequencies of alleles and genotypes of rs792243103 (–/C) in *PNKD* gene in 5 Polish breeds. | | | | | | |
| --- | --- | --- | --- | --- | --- | --- |
| Pig Breed | Frequencies of rs792243103 (–/C) in *PNKD* gene | | | | | |
|  | Genotype | | | Allele | | HWE*  (*P*-value) |
|  | CC | –/C | –/– | C | – |  |
| Polish Landrace | 0.22 (29) | 0.50 (66) | 0.28 (37) | 0.47 | 0.53 | 0.97 |
| Polish Large White | 0.60 (78) | 0.30 (40) | 0.10 (13) | 0.75 | 0.25 | 0.03 |
| Puławska | 0.34 (46) | 0.49 (67) | 0.17 (23) | 0.58 | 0.42 | 0.87 |
| Duroc | 0.53 (26) | 0.45 (22) | 0.02 (1) | 0.76 | 0.24 | 0.13 |
| Pietrain | 0.22 (20) | 0.56 (50) | 0.22 (20) | 0.50 | 0.50 | 0.29 |
| Total | 0.37 (199) | 0.46 (245) | 0.17 (94) | 0.60 | 0.40 |  |
| *HWE – Hardy-Weinberg Equilibrium: If p-value < 0.05 - not consistent with HWE, Not accurate if <5 individuals in any genotype group. In brackets number of individuals in genotype group. | | | | | | |
